# The first appearance of EEG evidence in a UK court of law: a cautionary tale

**DOI:** 10.1192/bjb.2022.88

**Published:** 2024-04

**Authors:** Ken Barrett

**Affiliations:** Uckfield, UK

**Keywords:** EEG, forensic, epilepsy, history of psychiatry, Grey Walter

## Abstract

Electroencephalogram-based evidence was accepted in a UK law court for the first time in 1939. This paper gives an account of that case, not previously clinically reported, and the individuals involved. Why it was not published in the literature at the time is explored and parallels with more recent technologies are highlighted.

In 1929 German psychiatrist Hans Berger published a paper describing rhythmic electrical pulsation of the brain that he named the ‘electroencephalogram’.^1^ His findings, initially considered ludicrous by the scientific establishment, were eventually confirmed in 1934 by Edgar Adrian, a Cambridge physiologist and Nobel Laureate who recorded his own alpha rhythm.^2^ Clinical electroencephalography (EEG) blossomed thereafter and the clinical value of the investigation, if not its functional significance, soon became clear.

Many papers explored the clinical use of the EEG in the decade after Adrian's paper, but only one, so far as I have been able to discover, concerned its use in a forensic setting:^3^ a crime of matricide, EEG being used as part of a defence based on a diagnosis of impaired judgement due to low blood sugar. The paper, authored by two psychiatrists, records the jury verdict of guilty but insane. Here I describe an earlier case, from 1939, not previously reported in the medical literature and almost certainly the first time EEG evidence was accepted in a UK law court. Details are drawn from police records, obtained through a request under the Freedom of Information Act 2000, accounts published in the press at the time (the trial was reported by all the broadsheets and several regional newspapers) and the unpublished autobiography of physiologist William Grey Walter.

## Background

Following the publication of Berger's initial report at least one UK clinician recognised the potential clinical value of the findings, should they be replicated. Frederick Golla was a neurologist at the National Hospital for Neurology and Neurosurgery (NHNN) in London and head of the Central Pathological Laboratory at the Maudsley Hospital.^4^ The latter role included oversight of laboratory investigations carried out for London's mental hospitals and of psychiatric research in the capital. After Adrian published his co-authored paper, Golla asked him if he could recommend a young scientist to work on the potential clinical value of the EEG. Adrian suggested a 25-year-old Cambridge scholar whose fellowship research had run into difficulties: William Grey Walter.^5^

Walter accepted the job and, after building his own EEG equipment, began recording at the Maudsley Hospital. Golla provided patients from the wards at the Maudsley and Walter soon demonstrated the alpha and other rhythms. In one patient, however, only slower rhythms were detected. Walter suspected a fault in his equipment until Golla revealed that the patient was not from the Maudsley but the NHNN, a patient believed to have a brain tumour.^5^ Following that finding, Walter and his equipment moved to the NHNN and, soon after, he published the first ever paper demonstrating the value of the EEG in localising brain tumours, followed by other papers, including studies of the EEG in epilepsy.^6^ He also gave talks on the new investigation at clinical meetings and following one such, in 1938, he was contacted by a psychiatrist, Dr L.A. Parry, who asked whether there was a portable version of the test equipment. He had a patient awaiting trial for murder whose defence rested on a diagnosis of epilepsy and EEG evidence might usefully support this. Having just built a portable version of his equipment, Walter agreed to make a recording in the prison (Natasha Walter, personal communication). He first sought permission from the Hospital Secretary, who agreed and, believing the story might interest the local press, contacted the *Daily Sketch*, one of London's large-circulation newspapers. A reporter and photographer were duly dispatched to interview Walter. The reporter agreed to be photographed having his EEG recorded and was duly impressed: ‘Mr Walter [ … ] showed me his almost magical brainwave machine yesterday in the little room in Maida Vale Nervous Diseases Hospital where he perfected his invention’. The resulting article, headlined ‘Mind-wave machine will be evidence first time in murder trial to-day’ went on to describe Walter as a ‘meteoric young scientist who has revolutionised brain healing’ and also suggested that the EEG was his discovery.^7^ The article was published on the first morning of the trial.

## The crime^8–10^

On 22 November 1938, JD, a 46-year-old unemployed ice-cream salesman, cycled to his allotment. On the way he was observed to speak to a young boy and girl, then cycle off with the 4-year-old girl, PO, perched on his crossbar. He later recalled ‘saluting a man and as I did so a blackness came over my eyes with terrible pain. I remember nothing more until I came to sitting on a seat with my head in my hands’. PO disappeared and 3 days later her body was discovered on the same allotments, partially covered by a blind. She had been strangled, but was otherwise unmolested. JD claimed to have no memory of the period before he returned to his senses, but he was the last person with whom she was seen and was charged with her murder. In view of the episode he described, his defence had him assessed by a local psychiatrist, Dr L.A. Parry. JD had been discharged from the army as medically unfit a year after joining up in 1914. He believed he suffered about 12 epileptic fits at that time. No previous psychiatric history was noted, but his uncle and an aunt had spent time in asylums. His defence subsequently rested on a diagnosis of epilepsy, the offence occurring during an ‘epileptiform episode’ in which his reason was impaired; his plea would be ‘guilty but insane’. Walter's EEG recording did, indeed, show the features seen in other people with epilepsy, with slowing over the frontal lobes, and so JD's defence team decided to use Walter as a witness for the defence.

## The trial

The trial began on 8 March 1939 at Lewes assizes and Mr Justice Charles, presiding, took some persuading to allow Walter's testimony, drawing analogy between the EEG and other inadmissible evidence based on ‘instruments supposed to tell if a man were telling the truth or lies’.^10^ Defence counsel conceded this was ‘rather unusual evidence’, continuing ‘but Mr Walter has used an instrument which, I believe, is useful to find an abnormality in a person’. ‘Used by a man who is totally unqualified,’ responded the judge (i.e. not a physician). Defence counsel replied that Walter was a physiologist and so qualified in the use of the new EEG machine. The EEG-based evidence was finally heard, but the judge then turned to the publication of the *Daily Sketch* article that morning, saying, as reported in *The Times*: ‘it was said to be an almost magical brain wave machine, as to seduce a jury, perhaps, into giving a verdict in accordance with the machine and against the true evidence’.^9^ He considered its publication, in advance of the trial, ‘a gross contempt of court’ and said he intended to cite Walter and the *Daily Sketch* editor with contempt. Only when the editor sent an abject apology the following day, accepting responsibility for the error, was the threat withdrawn. However, in a subsequent press interview the judge described the article itself as ‘a cheap puff for this Mr Walter. It attributes to him matters which when he came to set upon his oath he had to deny the truth of [ … ] it was invented by someone in Jena ten years ago and he has simply been dabbling away with it without any sort of medical information or education at all. He is simply a Master of Arts at Cambridge’.^10^

The outcome of the trial was recorded in the Metropolitan Police file:

‘in view of the medical history of epilepsy [ … ] in the absence of motive even our own witnesses were forced to admit that the only explanation was that the murder was committed by JD during a period described as “Epileptiform”, i.e. a period of complete irresponsibility followed by an entire absence of memory. After ten minutes the Jury returned a verdict of “Guilty but insane”, he was sentenced to be detained at His Majesty's pleasure and admitted to Broadmoor Hospital’.^8^

The Trial of Lunatics Act 1883 provided for the jury to return a verdict of ‘guilty of the act or omission charged, but insane as not to be responsible, according to law, for his action’, often abbreviated, as in this case, to ‘guilty but insane’. The Act was passed at the request of Queen Victoria, who had been the target of attacks by mentally ill individuals and demanded the previous ‘not guilty’ verdict be changed as a deterrent.^11^ The police file concludes with this comment from a senior officer: ‘I have read herein about the “mind machine” or the “brain tester” and the judge's remarks. I doubt whether we shall hear any more about this machine in subsequent murder cases’.^8^ JD remained in Broadmoor until his death in 1953. The case records are closed to the public until 2068, but were examined by hospital records staff, at my request, to determine whether the original EEG record or a report were present: neither was found.

## Walter's own account

Shortly after his court appearance Walter moved, with Golla, his chief, to a new post at the Burden Neurological Institute in Bristol, where he was to remain for the rest of his career. He died in 1977 and towards the end of his life wrote an unpublished autobiography which includes his recollections of the above events (I thank Natasha Walter for permission to quote from the autobiography here). On recording the EEG in Lewes prison he wrote:

‘I was horrified by the conditions in that prison: it was all stone, with dim gaslight. But my machine worked and I took a record. It was “abnormal” and suggested temporal lobe epilepsy, which fitted with the history.’

He goes on to describe

‘a very disagreeable incident [ … ] When I got to Lewes on the morning of the trial, there was a bunch of eager reporters: a London newspaper had published a crack story with the headline “Cambridge undergraduate tests murderer”. That was bad enough, but in the story there was a garbled account of my findings, made up from my publications and quite irrelevant. I had told none of my observations, just informed the Secretary of my absence … ’

Walter therefore appeared to forget that the article was based on an interview he gave to a journalist whose EEG he was photographed recording. It should be noted, however, that Walter suffered a severe head injury in 1969, which may have impaired his recall.^12^ On the trial, his account is more accurate:

‘I gave an account of my observations, but when I had finished the Judge glared at me, asked about my career and experience, then announced that he was going to arraign me for “contempt of court”, having disclosed my evidence in a popular newspaper.’

## Discussion

Over the years Walter gave many interviews to the media to promote his work and neuroscience more widely, including feature articles in newspapers and magazines.^13^ In the 1950s he gave a series of radio talks on the brain for the BBC that formed the basis of his book *The Living Brain*, one of the first books on the brain for the layperson.^5^ He later appeared on the TV panel show *The Brains Trust*, creating a public profile unusual for a scientist at that time, contributing to his reputation as an original thinker but also something of a self-publicising maverick.^13^

The introduction into the courtroom of evidence based on new technology remains problematic. This was recently illustrated, more than 70 years after the present case, by the forensic use of brain imaging-based lie detection.^14^ Sharing new technologies and their results with the popular press also continues to be fraught with difficulties, a recent journal article observing that, quite apart from reporter error or deliberate exaggeration, ‘competition, hyperspecialization, and the need to obtain funding for research projects might drive scientists to misrepresent their findings’.^15^

In March 1939, when this trial occurred, Walter was about to move to a new permanent post, as scientific director of the Burden Neurological Institute in Bristol, a new venture funded by a bequest from a Reverend Burden, a local healthcare entrepreneur.^4^ Far from enhancing his reputation, and that of the EEG, Walter's appearance in court lead to embarrassing articles in the national and regional press and a near arraignment for contempt of court. Neither would have endeared him to the charitable trust about to employ him. Small wonder, therefore, that he decided not to draw further attention to the case by publishing an account in the medical literature of the time.

## Data Availability

Data availability is not applicable to this article as no new data were created or analysed in this study.

## About the author

**Ken Barrett**, MB BS, MA, MD, retired neuropsychiatrist, Uckfield, UK.
